# Accelerated three-dimensional free-breathing first pass cardiac perfusion at 1.5T

**DOI:** 10.1186/1532-429X-15-S1-P42

**Published:** 2013-01-30

**Authors:** Mehmet Akcakaya, Tamer A Basha, Murilo Foppa, Kraig V Kissinger, Warren J Manning, Reza Nezafat

**Affiliations:** 1 Medicine, Beth Israel Deaconess Medical Center, Harvard Medical School, Boston, MA, USA; 2 Radiology, Beth Israel Deaconess Medical Center, Harvard Medical School, Boston, MA, USA

## Background

Perfusion CMR provides an important diagnostic tool for the detection of myocardial ischemia in patients with known or suspected CAD [1]. Conventionally, 2D multislice imaging with 2-4 short-axis slices is often used for evaluation of left ventricular perfusion. 3D CMR perfusion has been proposed for its superior coverage and higher SNR [2]. However, accelerated imaging is required for adequate spatio-temporal resolution. Recent studies in accelerated CMR have shown that utilizing local information in the imaging volume significantly improves reconstruction quality [3,4]. However, such highly accelerated k-t based techniques used for 3D perfusion [3] require breath-hold acquisitions, which may be difficult following physical stress or for some patients. In this study, we sought to develop a compressed-sensing (CS) based image reconstruction technique utilizing local imaging features for highly-accelerated 3D free-breathing perfusion CMR.

## Methods

9 patients (46.0±14.8 years; 6 males) underwent 3D perfusion CMR on a 1.5T Philips Achieva magnet. Images were acquired using a saturation-recovery GRE sequence (TR/TE/α=2.1/1.2 ms/20^o^). 7.5-fold random undersampling, with fully-sampled central k-space (11 × 3 in ky-kz) was used to acquire 8 slices at 2.3 × 2.3 × 10 mm^3^ resolution within a 250 ms imaging window.

In the proposed reconstruction technique, each dynamic was first reconstructed individually with total variation (TV) regularization, which were then used for utilizing local features as follows: 10×10 image patches were hypothesized to be slowly-varying across 5 consecutive heartbeats. The locality in each volume was used to capture how various anatomies move differently, whereas the locality in time was used to reduce temporal blurring artifacts due to free-breathing. Principal component (pc) bases were derived for each of these 10 × 10 × 1 × 5 overlapping image volumes. Then the distinct x-pc sparsity of these volumes was used for thresholding the image in an iterative B_1_-weighted technique. Comparison images were generated using dynamic-by-dynamic (DBD) TV reconstruction, reconstruction using a pc basis for the whole volume (x-pc), and zerofilling of the data. Images were then scored by a blinded reader (1=poor, 4=excellent).

## Results

**Figure 1:**
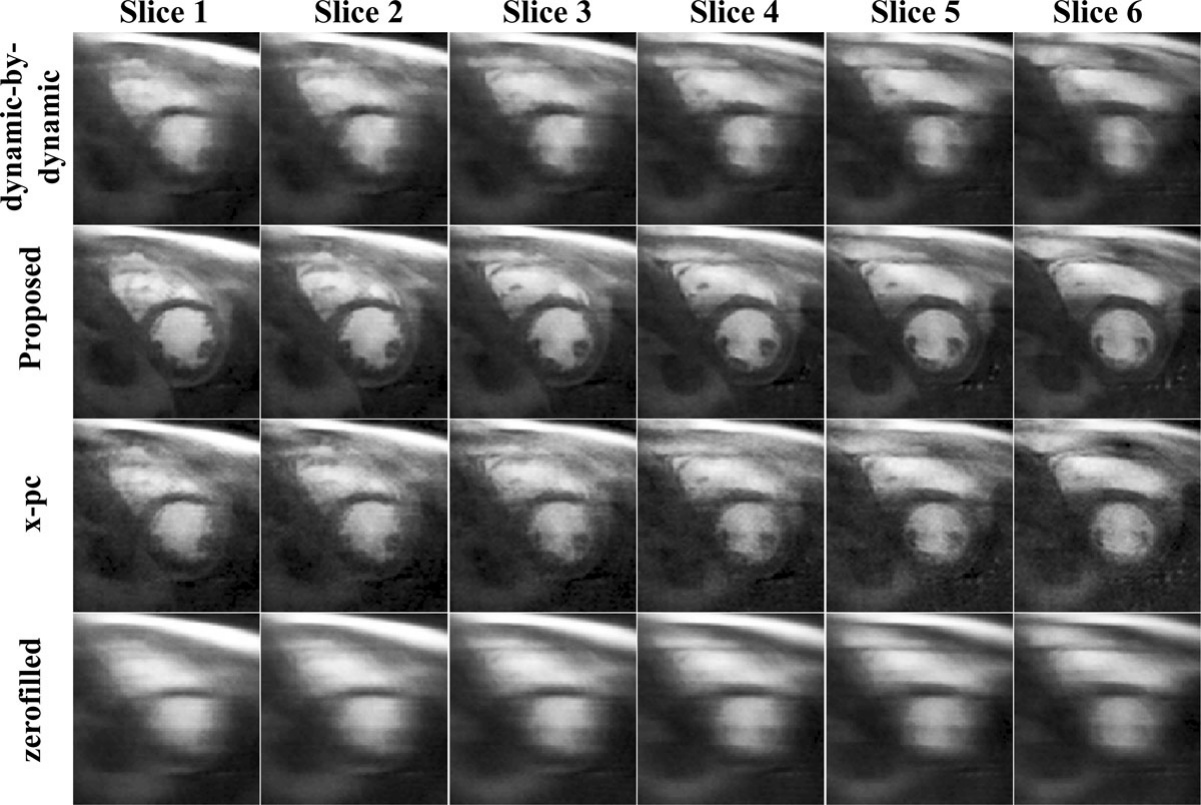
Example slices from a subject in a dynamic after the contrast arrival. Reconstructions using the proposed method have good temporal fidelity and are sharper compared to dynamic-by-dynamic CS reconstruction (top row), and x-pc CS reconstruction (3rd row), which suffer from blurring due to respiratory motion. The acquired (zerofilled) data is depicted in the bottom row.

**Figure 2:**
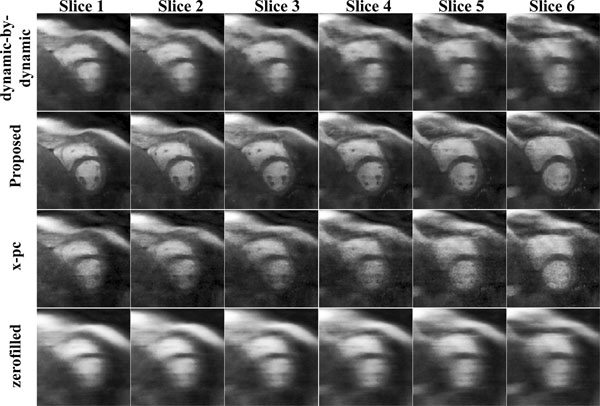
Example slices from another subject in a dynamic after the contrast arrival. Reconstruction from the proposed method have good temporal fidelity and are sharper compared to the dynamic-by-dynamic CS reconstruction (top row). The reconstructions with x-pc CS reconstruction (3rd row) suffer from blurring due to the breathing pattern in this dynamic and use of a single basis for the whole volume.

Figure 1 and 2 show example dynamics after contrast arrival in two subjects. The proposed technique is able to provide images with clearly defined borders, without temporal blurring. Subjective image quality scores demonstrate that the proposed method is significantly better than the others (3.0±0.7 vs. 2.3±0.7 for x-pc, 1.7±0.5 for DBD, 1.0±0.0 for zerofilled).

## Conclusions

The proposed framework allows for 7.5-fold accelerated free-breathing 3D perfusion reconstruction at 1.5T. Further clinical studies are needed to assess its diagnostic value in patients with suspected CAD.

## Funding

NIH:K99HL111410-01; R01EB008743-01A2.
